# Cigarette smoke alters the transcriptome of non-involved lung tissue in lung adenocarcinoma patients

**DOI:** 10.1038/s41598-019-49648-2

**Published:** 2019-09-10

**Authors:** Giulia Pintarelli, Sara Noci, Davide Maspero, Angela Pettinicchio, Matteo Dugo, Loris De Cecco, Matteo Incarbone, Davide Tosi, Luigi Santambrogio, Tommaso A. Dragani, Francesca Colombo

**Affiliations:** 1 Department of Research, Fondazione IRCCS Istituto Nazionale dei Tumori, Milan, Italy; 2 Platform of Integrated Biology, Department of Applied Research and Technology Development, Fondazione IRCCS Istituto Nazionale dei Tumori, Milan, Italy; 3 Department of Surgery, IRCCS MultiMedica, Sesto S.G., Italy; 4 Fondazione IRCCS Ca’ Granda Ospedale Maggiore Policlinico, Milan, Italy

**Keywords:** Transcriptomics, Molecular medicine

## Abstract

Alterations in the gene expression of organs in contact with the environment may signal exposure to toxins. To identify genes in lung tissue whose expression levels are altered by cigarette smoking, we compared the transcriptomes of lung tissue between 118 ever smokers and 58 never smokers. In all cases, the tissue studied was non-involved lung tissue obtained at lobectomy from patients with lung adenocarcinoma. Of the 17,097 genes analyzed, 357 were differentially expressed between ever smokers and never smokers (FDR < 0.05), including 290 genes that were up-regulated and 67 down-regulated in ever smokers. For 85 genes, the absolute value of the fold change was ≥2. The gene with the smallest FDR was *MYO1A* (FDR = 6.9 × 10^−4^) while the gene with the largest difference between groups was *FGG* (fold change = 31.60). Overall, 100 of the genes identified in this study (38.6%) had previously been found to associate with smoking in at least one of four previously reported datasets of non-involved lung tissue. Seven genes (*KMO*, *CD1A*, *SPINK5*, *TREM2*, *CYBB*, *DNASE2B*, *FGG*) were differentially expressed between ever and never smokers in all five datasets, with concordant higher expression in ever smokers. Smoking-induced up-regulation of six of these genes was also observed in a transcription dataset from lung tissue of non-cancer patients. Among the three most significant gene networks, two are involved in immunity and inflammation and one in cell death. Overall, this study shows that the lung parenchyma transcriptome of smokers has altered gene expression and that these alterations are reproducible in different series of smokers across countries. Moreover, this study identified a seven-gene panel that reflects lung tissue exposure to cigarette smoke.

## Introduction

Treatment of cell lines or laboratory animals with toxic chemicals alters their metabolism and induces a gene expression signature that reflects the effects of specific toxins or their mode of action. The analysis of alterations of gene expression of the whole transcriptome, following treatment with toxins, has originated a field of study called toxicogenomics^1,2^. The up- and down-regulated genes, following exposure to specific chemicals, are biomarkers of exposure but also provide evidence of the involved biochemical pathways and mechanisms of the toxic action^3^.

Tobacco combustion in cigarettes produces toxic compounds that have been shown to alter gene expression in directly exposed tissues, such as lung tissue^4^ and small airways^5^, and in indirectly exposed ones, such as blood^6,7^. In lung tissue, one study reported that cigarette smoke up-regulated cell cycle genes^4^. A more recent study^8^ identified 599 genes in lung tissue whose expression levels differed between never and current smokers, and while in former smokers these alterations had mostly normalized some genes remained up-regulated even 25 years after smoking cessation. Both these studies were carried out on non-involved lung tissue from patients with lung cancer, one of the most common diseases induced by tobacco smoking^9^. Smoking accounts for about 90% of all lung cancer cases^10^. Among the different lung cancer histotypes, lung adenocarcinoma is mainly observed in never smokers and former smokers^11^.

Understanding the molecular pathways in non-neoplastic lung tissue altered by smoking would provide clues to the pathogenicity of cigarette smoke. A way to reach this goal is identifying the signature of genes whose expression levels are modulated by cigarette smoking. We therefore performed a genome-wide transcriptome analysis on non-involved lung tissue from patients with lung adenocarcinoma, including 118 ever smokers and 58 never smokers, to investigate the differences in transcript levels and identify biochemical pathways altered by cigarette smoking.

## Results

### Lung tissue transcriptome distinguishes ever from never smokers

Microarray analysis of gene expression was done on 179 samples of non-involved lung tissue from 179 lung adenocarcinoma patients. For three samples (female ever smokers), microarray data were of poor quality, so these patients were excluded from analysis. Of the remaining 176 patients, 81 (46%) were female, 154 had pathological stage I cancer, and 58 were never smokers. The two groups of ever and never smokers had similar median ages at diagnosis: 66 years for ever smokers and 68 years for never smokers (Table 1). These two groups differed, instead, in the percentages of males and females, with relatively more females (71%) in the group of never smokers than in the ever smokers group (34%; *P* = 5.3 × 10^−6^, Fisher’s exact test). There was no significant association between group assignment and pathological stage (*P* = 0.57, Fisher’s exact test).

**Table 1:** Clinical characteristics of the 176 lung adenocarcinoma patients, by study group.

Characteristic	Ever smokers (n = 118)	Never smokers (n = 58)	*P*
Age at diagnosis, years, median (range)	66 (47–83)	68 (41–84)	0.9^§^
Sex, n			5.3 × 10^–6^*
*Female*	40	41	
*Male*	78	17	
Pathological stage, n			0.57 *
*I*	102	52	
*II*	6	4	
*III or IV*	9	2	
*Missing data*	1	0	
Dead at the 60-month follow-up, n (%)	18 (15.3)	7 (12.0)	0.80^^^

^§^Cox’s multivariable analysis, including sex as covariate, stratified by 10-year birth cohort. *Fisher’s exact test. ^^^Cox’s multivariable analysis, including sex, stage and age at diagnosis as covariates.

Microarray profiling provided data on 17,097 genes, including 15,525 protein-coding genes, 472 pseudo-genes, 742 non-coding RNAs, and 177 small nucleolar RNAs. Expression levels of these genes were compared between ever and never smokers to identify differentially expressed genes. Unsupervised clustering analysis of the samples according to their gene expression profiles did not distinguish the two groups (not shown). Instead, class comparison analysis revealed that 357 genes (2%) were differentially expressed with an FDR < 0.05 (Supplementary Table 1). Among these 357 genes, 290 were up-regulated and 67 were down-regulated in ever smokers. The absolute value of the fold change was ≥2 for 63 up-regulated and 22 down-regulated genes. The *FGG* gene had the widest difference in expression levels between groups (31.7-fold higher levels in ever than never smokers). The most down-regulated gene in ever smokers was *CDHR3*, whose levels were about 4-fold lower than those observed in never smokers.

**Figure 1:**
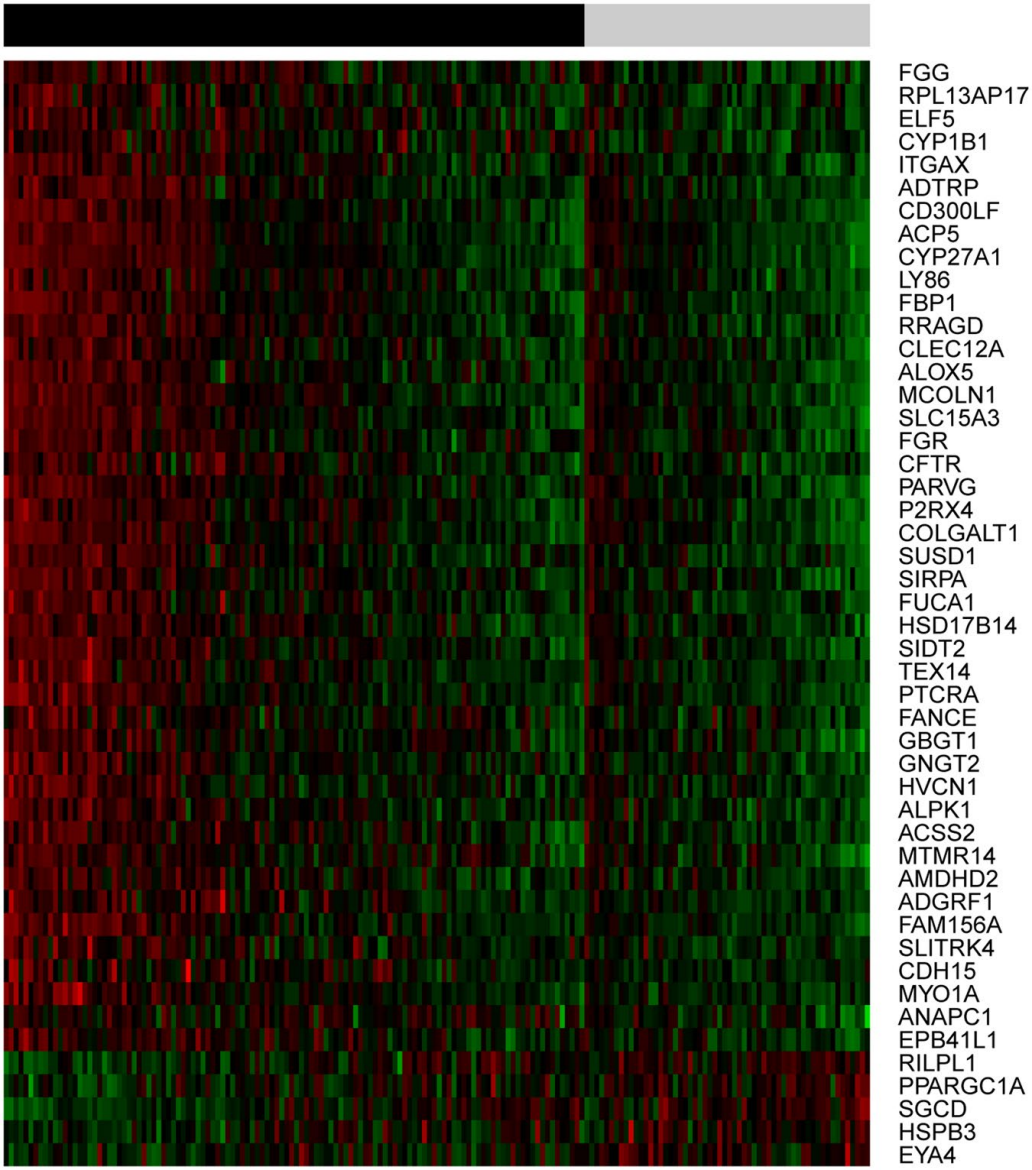
Expression levels of the 48 top-ranking differentially expressed genes in lung tissue between ever and never smokers. Results of the class comparison analysis (FDR < 0.01) are depicted as a heatmap with green indicating low expression and red high expression. Genes are ordered from top to bottom according to the expression fold change between the two groups. Across the top, the black bar marks ever smokers and the gray bar marks never smokers. Within these groups, samples are ordered according to the average expression of the genes.

At a statistical level of FDR < 0.01, there were 48 differentially expressed genes, and the top-ranking genes were *MYO1A* (FDR = 6.9 × 10^−4^, fold change = 1.42) and *RRAGD* (FDR = 6.9 × 10^−4^, fold change = 2.61), followed by *CYP1B1* (FDR = 7.4 × 10^−4^, fold change = 3.84). The expression levels of these 48 genes are illustrated in Fig. 1, where they are sorted according to fold change from 31.69 (*FGG*) to 0.40 (*EYA4*).

To estimate the cellular composition of the analyzed non-involved lung tissue samples, based on their transcriptome profiles, we used the bioinformatic tool xCell. This analysis found that, among 64 possible cell types, there was significant enrichment for two stromal cell types (i.e. lymphatic endothelial cells, and microvascular endothelial cells) and four immune cell types (i.e. dendritic cells, neutrophils, regulatory T-cells, and immature dendritic cells) in at least 80% of patients (Supplementary Fig. 1). Comparison of never smokers with ever smokers showed that the immune cell enrichment scores were similar (Supplementary Fig. 2A; *P* = 0.09, Kruskal-Wallis non-parametric test). In contrast, the stromal cell enrichment score was significantly lower in ever smokers than in never smokers (Supplementary Fig. 2B; *P* = 0.0036). However, for none of the six enriched cell types was there a significant difference in frequency between ever and never smokers (not shown).

### Cigarette smoke up-regulates inflammatory pathways

To identify molecular pathways in lung tissue affected by tobacco smoke, we analyzed the list of differentially expressed genes using the IPA online tool^12^. This analysis indicated that, among all the pathways defined in the IPA knowledge base, 52 were significantly associated with our gene dataset (Table 2). The top-ranking pathway was eicosanoid signaling (*P* = 1.7 × 10^−4^); seven of our differentially expressed genes encode proteins that participate in this pathway. Among the 52 identified pathways, 19 included at least five differentially expressed genes, with the sirtuin signaling pathway having the highest number of differentially expressed genes involved (n = 10). The majority of the identified pathways (n = 40) had differentially expressed genes that were all expressed at higher levels in ever smokers than in never smokers, suggesting that these pathways are up-regulated in people who smoke.

**Table 2:** Pathways enriched in genes differentially expressed in lung tissue, between ever and never smokers, according to Ingenuity Pathway Analysis. The 52 pathways are listed by increasing *P*-value.

Ingenuity canonical pathways	*P*-value^1^	Involved genes
Eicosanoid Signaling	1.7E-04	PLBD1, LTA4H, PLA2G4E, ALOX5AP, ALOX5, TBXAS1, DPEP2
Pentose Phosphate Pathway	9.1E-04	PGD, TALDO1, G6PD
UDP-N-acetyl-D-galactosamine Biosynthesis II	9.1E-04	HK2, GALE, HK3
D-glucuronate Degradation I	1.0E-03	AKR1A1, DCXR
Phagosome Maturation	1.0E-03	ATP6V0B, ATP6V1F, VPS33A, ATP6V0D1, CYBB, LAMP1, ATP6V1G2, ATP6AP1, ATP6V1B2
Mevalonate Pathway I	1.2E-03	ACAT2, HMGCR, HMGCS1
Superpathway of Cholesterol Biosynthesis	1.4E-03	FDFT1, ACAT2, HMGCR, HMGCS1
NAD biosynthesis II (from tryptophan)	1.5E-03	KMO, NADSYN1, QPRT
Leukotriene Biosynthesis	1.5E-03	LTA4H, ALOX5, DPEP2
TREM1 Signaling	1.8E-03	TREM1, GRB2, TYROBP, LAT2, NLRC4, ITGAX
Pentose Phosphate Pathway (Oxidative Branch)	2.1E-03	PGD, G6PD
Superpathway of Geranylgeranyldiphosphate Biosynthesis I (via Mevalonate)	2.9E-03	ACAT2, HMGCR, HMGCS1
GP6 Signaling Pathway	2.9E-03	BTK, COL8A2, COL21A1, GRB2, SYK, FCER1G, FGA, FGG
Trehalose Degradation II (Trehalase)	3.2E-03	HK2, HK3
Lipid Antigen Presentation by CD1	4.8E-03	CD1A, FCER1G, AP2S1
NAD Biosynthesis from 2-amino-3-carboxymuconate Semialdehyde	4.8E-03	NADSYN1, QPRT
Dendritic Cell Maturation	5.5E-03	IL1A, GRB2, TYROBP, CD1A, HLA-DMA, TREM2, FCER1G, IKBKE, IRF8
Phagosome Formation	6.8E-03	ITGB2, MSR1, GRB2, SYK, FCER1G, MARCO, ITGAX
CTLA4 Signaling in Cytotoxic T Lymphocytes	7.2E-03	PTPN6, GRB2, SYK, AP1S3, FCER1G, AP2S1
Atherosclerosis Signaling	7.6E-03	PLBD1, ITGB2, PLA2G4E, IL1A, MSR1, ALOX5, RBP4
FXR/RXR Activation	8.3E-03	PPARG, IL1A, CYP27A1, FBP1, FGA, RBP4, PPARGC1A
Tryptophan Degradation III (Eukaryotic)	9.3E-03	KMO, ACAT2, GCDH
GDP-glucose Biosynthesis	1.1E-02	HK2, HK3
Glucose and Glucose-1-phosphate Degradation	1.4E-02	HK2, HK3
Ketogenesis	1.4E-02	ACAT2, HMGCS1
NAD Phosphorylation and Dephosphorylation	1.4E-02	ACP5, NADK
Methylglyoxal Degradation VI	1.8E-02	LDHD
UDP-N-acetyl-D-galactosamine Biosynthesis I	1.8E-02	GALE
Androgen Biosynthesis	2.0E-02	HSD3B7, HSD17B14
Ethanol Degradation II	2.0E-02	AKR1A1, DHRS9, ACSS2
3-phosphoinositide Degradation	2.2E-02	PTPN6, PTPMT1, MTMR14, FIG. 4, EYA4, ACP5, SIRPA
Bile Acid Biosynthesis, Neutral Pathway	2.3E-02	CYP27A1, HSD3B7
Natural Killer Cell Signaling	2.3E-02	PTPN6, GRB2, TYROBP, SYK, FCER1G, SIGLEC7
LXR/RXR Activation	2.4E-02	FDFT1, IL1A, MSR1, FGA, HMGCR, RBP4
CD28 Signaling in T Helper Cells	2.5E-02	PTPN6, GRB2, HLA-DMA, SYK, FCER1G, IKBKE
Noradrenaline and Adrenaline Degradation	2.6E-02	MAOB, AKR1A1, DHRS9
Chondroitin Sulfate Degradation (Metazoa)	2.6E-02	GM2A, HEXB
Production of Nitric Oxide and Reactive Oxygen Species in Macrophages	2.6E-02	PTPN6, GRB2, CYBB, IKBKE, IRF8, NCF4, SIRPA, RBP4
PPAR Signaling	2.7E-02	PPARG, IL1A, GRB2, IKBKE, PPARGC1A
IL-8 Signaling	2.9E-02	MYL9, ITGB2, GRB2, CYBB, IKBKE, CSTB, EIF4EBP1, ITGAX
Dermatan Sulfate Degradation (Metazoa)	3.0E-02	GM2A, HEXB
Sirtuin Signaling Pathway	3.0E-02	PPARG, ATG7, SLC25A6, LDHD, NQO1, ACSS2, G6PD, TSPO, ATG16L2, PPARGC1A
Extrinsic Prothrombin Activation Pathway	3.4E-02	FGA, FGG
Glutaryl-CoA Degradation	3.4E-02	ACAT2, GCDH
Graft-versus-Host Disease Signaling	3.5E-02	IL1A, HLA-DMA, FCER1G
Acute Phase Response Signaling	3.6E-02	IL1A, HP, GRB2, IKBKE, FGA, FGG, RBP4
Epoxysqualene Biosynthesis	3.6E-02	FDFT1
Choline Degradation I	3.6E-02	CHDH
Taurine Biosynthesis	3.6E-02	CDO1
Cardiolipin Biosynthesis II	3.6E-02	PTPMT1
Intrinsic Prothrombin Activation Pathway	3.9E-02	KLK7, FGA, FGG
Role of NFAT in Regulation of the Immune Response	4.3E-02	BTK, GRB2, HLA-DMA, SYK, FCER1G, IKBKE, GNAZ

^1^Right-tailed Fisher’s exact test.

**Figure 2:**
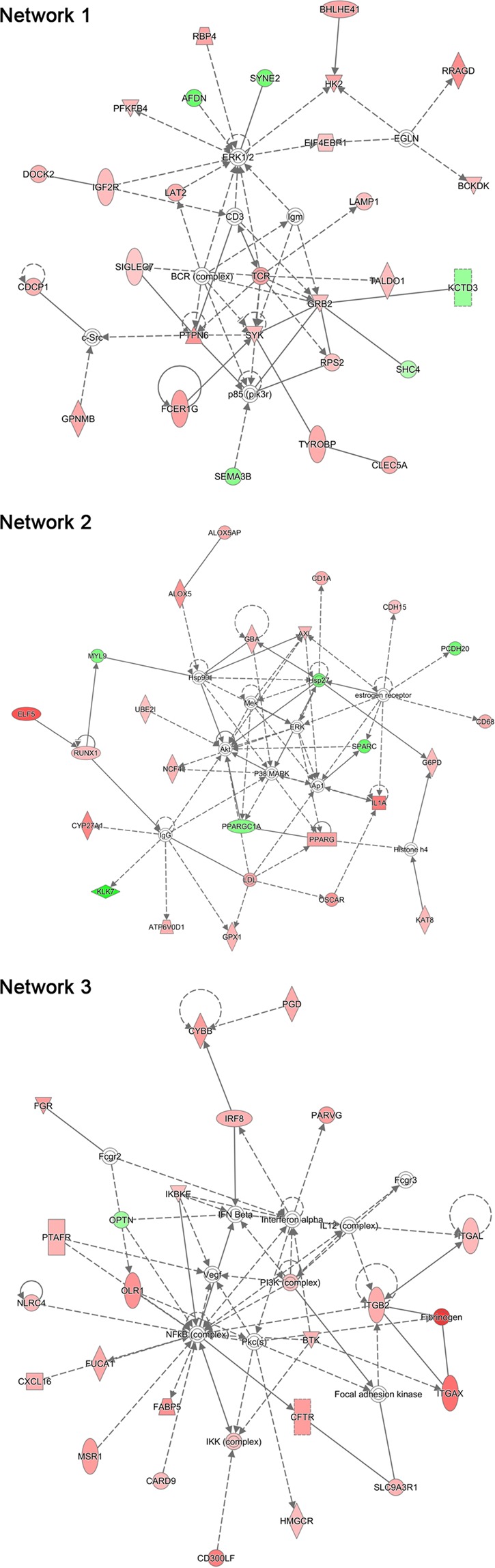
Illustration of the three networks that clustered the highest number of genes in lung tissue that were differentially expressed between ever and never smokers. Networks were identified using Ingenuity Pathway Analysis. Genes in red are up-regulated, while genes in green are down-regulated in ever smokers with respect to never smokers. Solid arrows indicate direct interactions, whereas dashed arrows indicate indirect interactions. The networks were generated through the use of IPA (QIAGEN Inc., https://www.qiagenbioinformatics.com/products/ingenuity-pathway-analysis).

The analysis of gene interactions identified 14 networks with score >5 (Supplementary Table 2). The biological processes that recurred the most were inflammation, cell interactions, and cell death and survival. Different networks had only a few genes in common. The three networks that clustered the highest number of genes are represented in Fig. 2. Network 1 is composed of genes that have a role in the regulation of cell death and survival, cellular compromise, cell-to-cell signaling and interaction; 27 of our differentially expressed genes are included in this network. The genes in Network 2 are involved in immunological disease, dermatological diseases and conditions, and inflammatory disease; this network includes 24 of our differentially expressed genes. Network 3, instead, is composed of genes that have a role in infectious diseases, regulation of cellular movement, and immune cell trafficking; 23 differentially expressed genes are in this network.

### Gene expression signatures overlap between studies

We next assessed the correspondence between our results and those from similar studies by Landi *et al*.^4^ and Bossé *et al*.^8^; this last study reported datasets from three independent series, hereafter called Laval, GRN and UBC. To this aim, we filtered our gene list (hereafter called IT) and the Laval, GRN and UBC lists according to the same criteria used by Landi *et al*. (*P*-value < 0.001 and absolute value of fold-change >1.5). As a result, we had 259 (IT), 591 (Laval), 113 (UBC), 129 (GRN), and 99 (Landi) genes for comparison.

**Figure 3:**
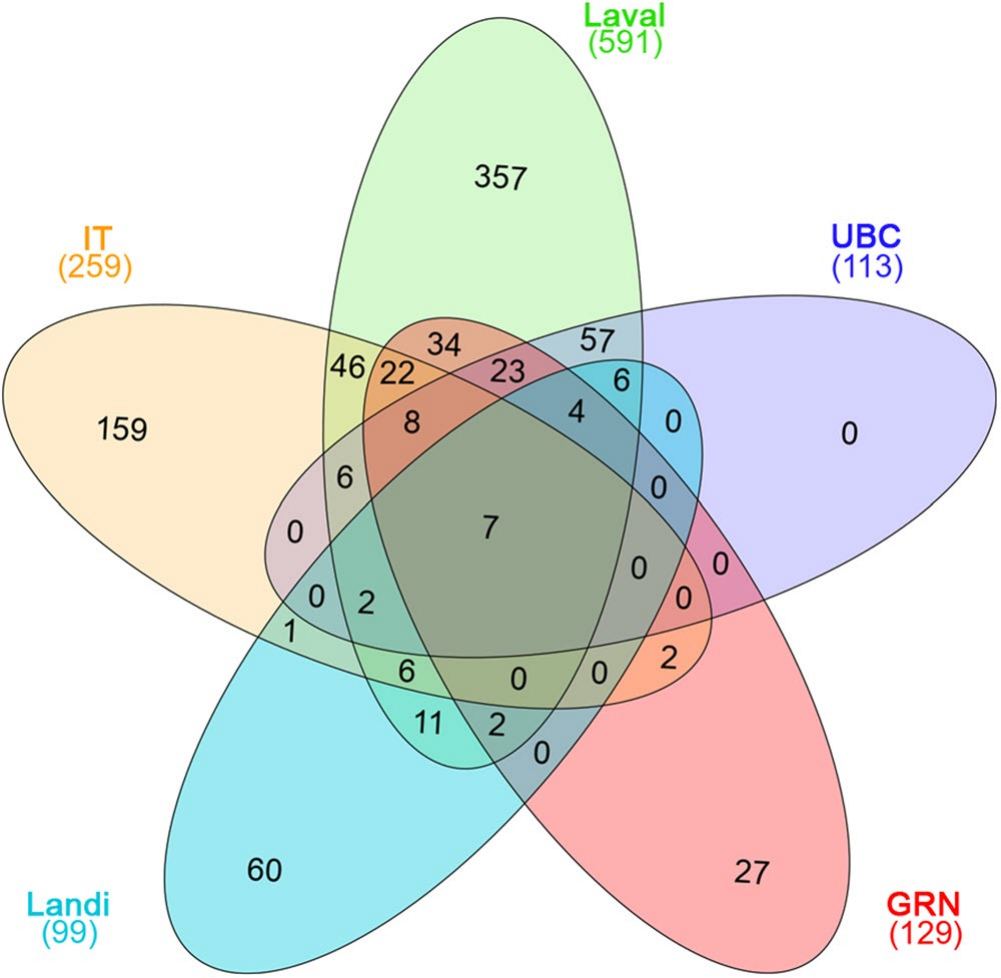
Intersection of the lists of genes significantly associated with smoking status in the five compared datasets. Venn diagram represents the genes differentially expressed between smokers (ever smokers in this study and current smokers in the other studies) and never smokers in non-tumor lung tissue with *P*-value < 0.001 and fold change >1.5. Each ellipse depicts the number of genes in each dataset from the various studies: IT (orange), present study; Laval (green), UBC (blue), and GRN (red), Bossé *et al*.^8^; and Landi (light blue), Landi *et al*.^4^. Numbers in the Venn diagram identify gene lists unique to each section. The total number of genes in each study is reported in brackets under the corresponding label.

The intersection of these five gene datasets is represented in the Venn diagram in Fig. 3. Overall, 100 of the 259 genes (38.6%) in the IT dataset were present in at least one of the other datasets, with 100% concordance in the direction of the effect of smoking on gene expression. In detail, 97, 23, 39 and 16 of the 259 IT genes were also differentially expressed in the Laval, UBC, GRN and Landi datasets, respectively. Interestingly, seven genes (*KMO*, *CD1A*, *SPINK5*, *TREM2*, *CYBB*, *DNASE2B*, *FGG*) were found to be differentially expressed between ever and never smokers in all five datasets, with concordant higher expression in ever smokers than in never smokers (Supplementary Table 3). Technical validation of the expression data of these seven genes was carried out by quantitative PCR in 54 patients from our series (27 ever and 27 never smokers). These data correlated positively with expression levels measured by microarray analysis (Supplementary Fig. 3). These seven genes were therefore considered to constitute a gene signature of smoking in non-involved lung tissue of patients with lung adenocarcinoma.

### The gene signature is not unique to lung tissue from cancer patients

To determine if the seven-gene signature of smoking is specific to lung cancer patients or is also valid in non-cancer patients, we obtained the GSE47460 dataset of gene expression in lung tissue from 254 patients with interstitial lung disease, 220 patients with chronic obstructive lung disease (COPD), and 108 persons who had surgery for a suspicious lung nodule but were found to not have any lung disease. Of these 582 non-cancer cases, 23 were classified as current smokers, 400 as former smokers, and 136 as never smokers; for the remaining 23 cases information on smoking habit was not available.

Class comparison analysis was used to identify differentially expressed genes between: (i) ever smokers (current and former smokers, n = 423) and never smokers; and (ii) current and never smokers. These analyses identified nine and 1160 differentially expressed genes, respectively, at FDR < 0.05 (not shown). Examination of the first gene list (ever vs. never smokers) revealed that one of our seven signature genes, *FGG*, was also upregulated in ever smokers without cancer (Supplementary Table 4). Examination of the second gene list (current vs. never smokers) revealed that all genes of our signature of smoking, except *SPINK5*, were upregulated in current smokers without cancer. This analysis suggests that the seven-gene signature of smoking is not unique to lung cancer patients.

## Discussion

This study compared the transcriptional landscape of non-involved lung tissue between ever and never smokers surgically treated for lung adenocarcinoma. We found that 357 genes significantly associated with smoking habit. Most of them were involved in biological processes such as inflammation, cell interactions, and cell death and survival. Almost 40% of our gene set overlapped with genes found to be differentially expressed in at least one of four published datasets^4,8^. Moreover, the effect of smoking on the transcription of these 100 genes was concordant between our study and the others, namely smoking always induced their up-regulation. Seven of these genes are up-regulated in five datasets, from this study and two others^4,8^.

Most of the genes that we found to be differentially expressed between ever and never smokers participate in pathways or networks that are associated with immunity, inflammation, or cell survival processes. This is congruent with an alteration of the protective mechanisms of the lung tissue induced by the toxic effect of cigarette smoking^13^. Of note, the most significantly enriched pathway was that of eicosanoid signaling, which produces lipids such as prostaglandins and leukotrienes that are important in initiating inflammation^14^. Up-regulation in lung tissue of genes in the eicosanoid pathway is concordant with the pro-inflammatory effects of smoke^15^ and is supported by experimental evidence. One study showed that human lung fibroblasts, when treated in *vitro* with cigarette smoke extract, up-regulated expression of *COX2* (the gene that encodes cyclooxygenase 2), leading to high levels of prostaglandin E2^16^. Two longitudinal studies of volunteers on a smoking-cessation program found that cigarette smoking increased systemic eicosanoid synthesis, resulting in abnormally high urine levels, and that these levels normalized upon smoking cessation (in those volunteers who did not use nicotine replacement therapy)^17,18^.

Among the seven genes found to be up-regulated by smoke in our study and in two previous reports^4,8^ is *KMO*, which codes for mitochondrial kynurenine 3-monooxygenase. This enzyme eliminates kynurenine, a byproduct of tryptophan metabolism, and thereby prevents its conversion to kynurenic acid^19^. This latter molecule was shown to be an antagonist of the α7 nicotinic acetylcholine receptor^20^, which is involved in the rewarding effects of nicotine^21^. It has recently been demonstrated that inhibition of kynurenine 3-monooxygenase decreased nicotine self-administration in animal models^22^. Our finding of a significant up-regulation of *KMO* gene in lung tissue of smokers is in agreement with these in *vivo* data, and suggests that the KMO enzyme might be a biomarker of smoking addiction in the lungs of smokers.

Another gene up-regulated in our study and the two previous studies is *TREM*2, which encodes TREM-2, a protein involved in tyrosine kinase-mediated membrane signaling. A recent preclinical study suggested that TREM-2 might be a negative immune regulator in lung cancer^23^.

The other five genes of our signature currently have only limited relevance to lung cancer. *DNASE2B*, which is part of the DNase II family of DNases, is expressed in the lung^24^; however, its role in lung cancer is not yet characterized. *FGG*, which encodes the gamma chain of fibrinogen, is down-regulated in the epithelial-to-mesenchymal transition, an important mechanism in cancer metastasis^25^. *CYBB* encodes the beta chain of cytochrome B-245; germline mutations in this gene are associated with chronic granulomatous disease, a hereditary immunodeficiency syndrome^26^. *CD1A*, which codes for a member of the CD1 transmembrane proteins, is abundantly expressed on Langerhans cells and is involved in inflammatory skin diseases^27^. *SPINK5* encodes a serine peptidase inhibitor; germline missense mutations in this gene have been associated with allergic conditions and atopic manifestations^28^.

Besides these seven genes, one other gene was found to be up-regulated in ever smokers in our study and in the two previous studies^4,8^, but below statistical threshold in the GRN series. This gene is *CYP1B1*, encoding cytochrome P450 family 1 subfamily B member 1. *CYP1B1*, together with *CYP1A1*, *CYP3A4* and *CYP3A5*, is involved in the metabolism of pro-carcinogens contained in cigarette smoke. The induction of *CYP1B1* expression may be due to a feedback mechanism stimulated by exposure to cigarette smoke^29^.

Several studies have already documented the effects of cigarette smoking on lung gene transcription in otherwise healthy persons and in patients with various lung diseases. Analysis of one published dataset of gene expression in lung tissue from non-cancer patients (GSE47460) allowed us to validate six of the seven genes of our signature of smoking as being up-regulated in current smokers even in the absence of a diagnosis of cancer. This result indicates that this gene panel is not unique to patients with lung cancer. Because the number of current smokers in GSE47460 is small (n = 23), further studies of non-cancer patients are needed. Smoke-induced changes in gene expression have also been found in small airway epithelium^29–31^, but none of the smoking-responsive genes identified in those studies are part of our seven-gene signature. This discrepancy may be due to the known differences in transcriptome profiles between bronchial epithelial cells and lung parenchyma^32^, and suggests that our gene panel is particular to the effects of smoke on lung parenchyma.

In the comparison of our data with those of Bossé *et al*.^8^ and Landi *et al*.^4^, differences were noted in the microarray platforms, covariates in the statistical analyses, and tumor histotype of patients whose non-involved lung tissue was analyzed (we and Landi *et al*. included only adenocarcinoma patients whereas Bossé *et al*. included patients with other lung cancer histotypes). These other studies compared the transcriptomes between never, former, and current smokers. Unfortunately we could not distinguish between current and former smokers, and so classified them as ever smokers. This difference is an interesting topic for future study, considering that it has been postulated that the long-term risk of developing several smoke-related diseases after smoking cessation is due to an alteration of epigenetic mechanisms that in turn could modify gene expression^33^. However, two recent epigenome-wide studies of lung tissue did not find differences in methylated CpG islands between smokers and non-smokers at any of the seven genes whose expression in non-involved lung tissue we found to associate with smoking status^34,35^.

A possible limitation of our study is the unequal group sizes, which can be attributed to the fact that most lung cancer patients are smokers or former smokers. Although we studied fewer never smokers than ever smokers, our group of never smokers is relatively large compared to that of similar studies^4,8^.

Among the genes differentially expressed in the non-involved lung tissue between ever and never smokers with lung cancer, we observed an up-regulation of genes expressed in immune system cells. This finding is congruent with the inflammatory condition that characterizes the lung tissue of smokers, and suggests that anti-inflammatory agents may reduce smoking-induced damage to lung tissue and, consequently, the risk of smoking-induced diseases. However, ever smokers taking non-steroidal anti-inflammatory drugs experience only a modest reduction of lung cancer risk that is apparent only in men^36,37^.

Overall, this study builds on two previous studies to identify a seven-gene panel that should be a solid and reproducible biomarker of smoking effects on lung tissue. This tool should facilitate the development and monitoring (in animal or in *vitro* models) of novel therapies that, by contrasting smoking effects, may be able to reduce the risk of cancer and of other diseases associated with smoking habit.

## Methods

### Population series and biological material

The study analyzed data and tissue samples from 179 patients who had lobectomy for lung adenocarcinoma at the Fondazione IRCCS Istituto Nazionale dei Tumori, Fondazione IRCCS Ca’ Granda Ospedale Maggiore Policlinico, and Ospedale San Giuseppe (all in Milan, Italy), between 1992 and 2017. The Committees for Ethics of the of the institutes involved in recruitment (Fondazione IRCCS Istituto Nazionale dei Tumori, Fondazione IRCCS Ca’ Granda Ospedale Maggiore Policlinico, and Ospedale San Giuseppe) approved the protocol for collecting samples and clinical data. Written informed consent had been obtained from the patients at recruitment to use their biological material and data for research purposes. The research was conducted in accordance with the tenets of the Declaration of Helsinki. All methods were performed in accordance with the relevant guidelines and regulations.

From each patient, we obtained a sample of non-involved (apparently normal) lung tissue collected as far as possible from the tumor site, i.e., from the so-called left-over material of surgical treatment. Tissue samples were flash-frozen or immersed in RNA*later* stabilization solution (Thermo Fisher Scientific) and stored at −80 °C or −20 °C, respectively. Clinical data for each patient regarded sex, age at diagnosis, pathological stage and self-reported habit regarding the smoking of tobacco-containing cigarettes. Smoking habit was recorded as either “ever” if they had ever smoked in their life (independently of whether they were currently smoking) or “never” if they had never smoked in their life.

### Gene expression analysis

Total RNA was extracted from lung tissue and subjected to gene expression profiling on Illumina HumanHT-12 v4 Expression BeadChips as previously reported^38^. Microarray raw data were log_2_-transformed and normalized using the robust spline normalization method implemented in the lumi Bioconductor package^39^. Normalized data were additionally adjusted for batch effects using ComBat^40^. Corrected data were then collapsed from probe level to gene level by selecting, for each gene, the probe with the highest mean intensity across samples. Finally, the dataset was filtered by removing genes not expressed in any samples.

Unsupervised clustering analysis was performed using hierarchical clustering considering the top-ranking 1500, 1000, and 500 variable genes across samples according to inter-quartile range. Hierarchical clustering was applied using 1-Pearson’s correlation coefficient as distance metric and average linkage parameter. In a class comparison analysis, genes that were differentially expressed between ever smokers and never smokers were identified using the linear modeling approach with empirical Bayes moderation implemented in the limma package^41^. This analysis was performed considering sex as a covariate. Heatmaps of differentially expressed genes were made using the heatmap.2 function of the gplots package.

*P*-values were corrected for multiple testing using the Benjamini-Hochberg false discovery rate (FDR) method. Genes with an FDR < 0.05 were considered differentially expressed. Fold change in gene expression was calculated as the ratio of ever smokers to never smokers. Gene expression data were deposited in the Gene Expression Omnibus database (GEO, http://www.ncbi.nlm.nih.gov/geo/) with accession number GSE123352.

### xCell analysis

The cellular composition of lung tissue samples was estimated from gene expression data using the online tool xCell tool using default parameters^42^ (http://xcell.ucsf.edu/). The tool produces, for each input sample (in this study, one transcriptome per patient), a score of enrichment for 64 different cell types. It also generates an immune cell enrichment score (considering B cells, CD4+ T-cells, CD8+ T cells, dendritic cells, eosinophils, macrophages, monocytes, mast cells, neutrophils, and NK cells) and a stromal cell enrichment score (considering adipocytes, endothelial cells, and fibroblasts). It also gives a *P*-value for the enrichment scores, which we set as <0.05 for the significance threshold. Normalized cell type enrichment scores (calculated, for each cell type, as the ratio between the enrichment score of each patient and the maximum value among all patients) were plotted using Heatmap3 in R environment. Enrichment scores were compared between never and ever smokers, for cell types having significant enrichment values in at least 80% of patients and for immune cells and stromal cells, using the Kruskal-Wallis test.

### Pathway analysis

To identify biological pathways whose activity could be altered by the effects of smoking on gene transcription, we used the Ingenuity Pathway Analysis (IPA) web-based software (Qiagen)^12^ with default parameters and selecting Human HT-12v4.0 array as the platform. Pathways were considered significantly affected if a right-tailed Fisher’s exact test *P*-value was <0.05. Networks were considered relevant if they had a score >5.

### Study comparison

To compare our transcriptome results with those of similar previous studies, we downloaded Supplementary Table 1 of Bossé *et al*.^8^ (http://cancerres.aacrjournals.org/content/72/15/3753) and Supplementary Table 3 of Landi *et al*.^4^ (http://dx.plos.org/10.1371/journal.pone.0001651). Both tables report fold changes in gene transcription between current and never smokers in non-involved lung tissue from lung cancer patients. Only those transcripts for which a gene symbol was available were considered here. Because Landi *et al*.^4^ reported data filtered on the basis of a *P*-value < 0.001 and an absolute value of fold change >1.5, we applied the same criteria to our results and to those of Bossé *et al*.^8^ in order to have comparable data for analysis. Venn diagrams were drawn using the InteractiVenn online tool^43^.

To assess the generalizability of our findings to lung tissue outside of the setting of lung cancer, we downloaded the normalized gene expression data from GSE47460 dataset. We carried out class comparison analyses as described above in the section “Gene expression analysis”, using sex and disease type (interstitial lung disease, COPD, or none) as covariates. This analysis was done between ever and never smokers, and between current and never smokers.

### Quantitative PCR

To technically validate the expression levels of the seven genes found to be differentially expressed in this study and in those of Bossé *et al*. and Landi *et al*.^4,8^, we did quantitative PCR in a subset of 54 patients (27 ever smokers and 27 never smokers) from this study. Total RNA (400 ng) was reverse-transcribed to cDNA using Superscript IV Vilo Master Mix (Thermo Fisher Scientific) and analyzed using TaqMan Gene Expression Assays (*KMO*: Hs00175738_m1, *CD1A*: Hs00381751_m1, *SPINK5*: Hs00199260_m1, *TREM2*: Hs00219132_m1, *CYBB*: Hs00166163_m1, *DNASE2B*: Hs00998752_m1, *FGG*: Hs00241037_m1, Thermo Fisher Scientific) and TaqMan Fast Advanced Master Mix (Thermo Fisher Scientific). Each reaction used 20 ng cDNA as template in a final volume of 10 ul. The human hypoxanthine phosphoribosyltransferase 1 (*HPRT1*: Hs99999909_m1) gene was used to normalize expression data. Reactions were run in duplicate on an ABI 7900HT platform (Life Technologies). Relative quantities of mRNA levels were assessed using the comparative cycle threshold (Ct) method and calculated with respect to an RNA sample extracted from non-involved lung tissue of a single patient, used as calibrator.

### Statistical analyses

Clinical characteristics between ever and never smokers were compared as follows: for age at diagnosis (a time-to-event variable), we used multivariable Cox proportional hazard regression, with sex as covariate, stratifying by decade of birth year^44^. For sex and pathological stage, Fisher’s exact test was used. Correlations between expression levels determined by microarray and by quantitative PCR were evaluated with Pearson’s test. xCell enrichment scores were compared using the non-parametric Kruskal-Wallis test. All statistical analyses were carried out using R software. A value of *P* < 0.05 was taken to indicate statistical significance.

## Supplementary information

Supplementary Table 1

Supplementary Table 2

Supplementary Table 3

Supplementary Table 4

Supplementary Figure 1

Supplementary Figure 2

Supplementary Figure 3
