# Supplementary material for: Cigarette smoke alters the transcriptome of non-involved lung tissue in lung adenocarcinoma patients
Source: Sci Rep. 2019 Sep 10;9:13039. doi: 10.1038/s41598-019-49648-2 (PMC6736939; doi:10.1038/s41598-019-49648-2)
Supplement: Supplementary file 1 — Supplementary Table 1 [file 41598_2019_49648_MOESM1_ESM.pdf]

Cigarette smoke alters the transcriptome of non-involved lung tissue in lung adenocarcinoma patients

Giulia Pintarelli, Sara Noci, Davide Maspero, Angela Pettinicchio, Matteo Dugo, Loris De Cecco, Matteo Incarbone, Davide Tosi, Luigi Santambrogio, Tommaso A. Dragani, Francesca Colombo

Supplementary Table 1. Genes differentially expressed between ever smokers and never smokers (FDR <0.05). The 357 genes are listed in order of increasing FDR

Gene symbol	Gene name	Chr.	Fold change[a]	FDR[b]
MYO1A	myosin IA	12	1.42	0.00069
RRAGD	Ras related GTP binding D	6	2.61	0.00069
CYP1B1	cytochrome P450 family 1 subfamily B member 1	2	3.84	0.00074
FGG	fibrinogen gamma chain	4	31.69	0.00133
MCOLN1	mucolipin 1	19	2.38	0.00227
CD300LF	CD300 molecule like family member f	17	2.97	0.00252
SIRPA	signal regulatory protein alpha	20	1.98	0.00252
ITGAX	integrin subunit alpha X	16	3.23	0.00252
CYP27A1	cytochrome P450 family 27 subfamily A member 1	2	2.77	0.00252
SUSD1	sushi domain containing 1	9	2.02	0.00252
EPB41L1	erythrocyte membrane protein band 4.1 like 1	20	1.33	0.00252
RPL13AP17	ribosomal protein L13a pseudogene 17	7	4.34	0.00252
TEX14	testis expressed 14, intercellular bridge forming factor	17	1.88	0.00252
PTCRA	pre T-cell antigen receptor alpha	6	1.85	0.00252
P2RX4	purinergic receptor P2X 4	12	2.05	0.00252
ALPK1	alpha kinase 1	4	1.67	0.00252
EYA4	EYA transcriptional coactivator and phosphatase 4	6	0.40	0.00336
RILPL1	Rab interacting lysosomal protein like 1	12	0.60	0.00336
SLC15A3	solute carrier family 15 member 3	11	2.34	0.00336
ELF5	E74 like ETS transcription factor 5	11	3.92	0.00341
FANCE	Fanconi anemia complementation group E	6	1.83	0.00355
FUCA1	alpha-L-fucosidase 1	1	1.96	0.00370
GBGT1	globoside alpha-1,3-N-acetylgalactosaminyltransferase 1 (FORS blood group)	9	1.77	0.00383
GNGT2	G protein subunit gamma transducin 2	17	1.69	0.00383
ADGRF1	adhesion G protein-coupled receptor F1	6	1.56	0.00425
ANAPC1	anaphase promoting complex subunit 1	2	1.41	0.00437
PARVG	parvin gamma	22	2.23	0.00477
HSPB3	heat shock protein family B (small) member 3	5	0.48	0.00477

HSD17B14	hydroxysteroid 17-beta dehydrogenase 14	19	1.90	0.00526
SLITRK4	SLIT and NTRK like family member 4	X	1.53	0.00598
CLEC12A	C-type lectin domain family 12 member A	12	2.53	0.00605
ACSS2	acyl-CoA synthetase short chain family member 2	20	1.65	0.00605
SIDT2	SID1 transmembrane family member 2	11	1.90	0.00605
MTMR14	myotubularin related protein 14	3	1.59	0.00605
ALOX5	arachidonate 5-lipoxygenase	10	2.44	0.00605
FGR	FGR proto-oncogene, Src family tyrosine kinase	1	2.29	0.00605
SGCD	sarcoglycan delta	5	0.48	0.00706
FBP1	fructose-bisphosphatase 1	9	2.72	0.00710
COLGALT1	collagen beta(1-O)galactosyltransferase 1	19	2.03	0.00770
CDH15	cadherin 15	16	1.49	0.00770
ACP5	acid phosphatase 5, tartrate resistant	19	2.84	0.00770
LY86	lymphocyte antigen 86	6	2.73	0.00772
PPARGC1A	PPARG coactivator 1 alpha	4	0.54	0.00777
AMDHD2	amidohydrolase domain containing 2	16	1.57	0.00844
FAM156A	family with sequence similarity 156 member A	X	1.56	0.00862
HVCN1	hydrogen voltage gated channel 1	12	1.69	0.00969
ADTRP	androgen dependent TFPI regulating protein	6	3.14	0.00969
CFTR	cystic fibrosis transmembrane conductance regulator	7	2.25	0.00969
CAP2	cyclase associated actin cytoskeleton regulatory protein 2	6	0.52	0.01017
CD1A	CD1a molecule	1	1.93	0.01017
ITPA	inosine triphosphatase	20	1.55	0.01017
ZNF589	zinc finger protein 589	3	2.04	0.01017
EIF4EBP1	eukaryotic translation initiation factor 4E binding protein 1	8	1.28	0.01017
PRSS21	protease, serine 21	16	2.79	0.01064
ATP6V0B	ATPase H+ transporting V0 subunit b	1	1.79	0.01075
FRMD3	FERM domain containing 3	9	0.53	0.01075
SMCO4	single-pass membrane protein with coiled-coil domains 4	11	1.91	0.01075
HK3	hexokinase 3	5	2.33	0.01075
FKBP15	FK506 binding protein 15	9	1.79	0.01075
SYNE2	spectrin repeat containing nuclear envelope protein 2	14	0.48	0.01075
PTPN6	protein tyrosine phosphatase, non-receptor type 6	12	2.45	0.01075
MMD	monocyte to macrophage differentiation associated	17	1.70	0.01075
ALOX5AP	arachidonate 5-lipoxygenase activating protein	13	1.95	0.01075
SLC1A7	solute carrier family 1 member 7	1	1.59	0.01075

BTK	Bruton tyrosine kinase	X	1.74	0.01075
DPEP2	dipeptidase 2	16	1.81	0.01075
GPR137B	G protein-coupled receptor 137B	1	1.71	0.01075
ELOVL7	ELOVL fatty acid elongase 7	5	0.68	0.01075
GCHFR	GTP cyclohydrolase I feedback regulator	15	3.04	0.01075
SLC37A2	solute carrier family 37 member 2	11	1.79	0.01075
SPINK5	serine peptidase inhibitor, Kazal type 5	5	2.12	0.01075
PCDH20	protocadherin 20	13	0.48	0.01075
COL21A1	collagen type XXI alpha 1 chain	6	0.61	0.01075
TMEM131L	transmembrane 131 like	4	1.56	0.01075
KLK7	kallikrein related peptidase 7	19	0.31	0.01075
SLITRK6	SLIT and NTRK like family member 6	13	0.76	0.01113
ATP6AP1	ATPase H+ transporting accessory protein 1	X	1.87	0.01164
TMEM91	transmembrane protein 91	19	1.86	0.01170
ATP6V1F	ATPase H+ transporting V1 subunit F	7	1.81	0.01170
RBP4	retinol binding protein 4	10	2.08	0.01179
HTRA4	HtrA serine peptidase 4	8	2.27	0.01183
TMEM251	transmembrane protein 251	14	1.75	0.01183
OSCAR	osteoclast associated, immunoglobulin-like receptor	19	2.46	0.01263
LAPTM5	lysosomal protein transmembrane 5	1	2.00	0.01263
DCSTAMP	dendrocyte expressed seven transmembrane protein	8	2.76	0.01263
IL1A	interleukin 1 alpha	2	3.09	0.01263
GPNMB	glycoprotein nmb	7	1.99	0.01263
HES2	hes family bHLH transcription factor 2	1	1.78	0.01284
JAML	junction adhesion molecule like	11	1.96	0.01284
RPS2	ribosomal protein S2	16	1.44	0.01320
TMC6	transmembrane channel like 6	17	1.87	0.01345
DOCK2	dedicator of cytokinesis 2	5	1.80	0.01351
EVI2B	ecotropic viral integration site 2B	17	2.37	0.01371
GUSB	glucuronidase beta	7	1.66	0.01371
PIAS2	protein inhibitor of activated STAT 2	18	1.42	0.01371
GLMP	glycosylated lysosomal membrane protein	1	1.69	0.01399
HMGCS1	3-hydroxy-3-methylglutaryl-CoA synthase 1	5	2.13	0.01402
KLHL6	kelch like family member 6	3	1.68	0.01402
IGF2R	insulin like growth factor 2 receptor	6	1.51	0.01425
HEXB	hexosaminidase subunit beta	5	1.66	0.01433
FDFT1	farnesyl-diphosphate farnesyltransferase 1	8	1.53	0.01503
ITGB2	integrin subunit beta 2	21	1.89	0.01568
COL8A2	collagen type VIII alpha 2 chain	1	1.59	0.01581
LTA4H	leukotriene A4 hydrolase	12	1.84	0.01589
PPM1M	protein phosphatase, Mg2+/Mn2+ dependent 1M	3	1.52	0.01589

ATP6V1B2	ATPase H+ transporting V1 subunit B2	8	1.47	0.01589
RENBP	renin binding protein	X	1.97	0.01648
TFPT	TCF3 fusion partner	19	1.72	0.01648
FGA	fibrinogen alpha chain	4	4.67	0.01694
SLC2A9	solute carrier family 2 member 9	4	1.61	0.01694
ago-04	argonaute 4, RISC catalytic component	1	1.33	0.01714
TSPAN8	tetraspanin 8	12	0.47	0.01798
ELOA	elongin A	1	1.53	0.01798
TSPAN10	tetraspanin 10	17	1.42	0.01810
OLR1	oxidized low density lipoprotein receptor 1	12	2.46	0.01830
GALE	UDP-galactose-4-epimerase	1	1.47	0.01838
HLA-DMA	major histocompatibility complex, class II, DM alpha	6	1.79	0.01840
SLC9A3R1	SLC9A3 regulator 1	17	1.76	0.01850
BIRC7	baculoviral IAP repeat containing 7	20	1.61	0.01868
UBIAD1	UbiA prenyltransferase domain containing 1	1	1.43	0.01872
NEXN	nexilin F-actin binding protein	1	0.53	0.01872
FABP5	fatty acid binding protein 5	8	2.48	0.01872
FIG4	FIG4 phosphoinositide 5-phosphatase	6	1.74	0.01872
LAT2	linker for activation of T-cells family member 2	7	1.87	0.01883
PRR13	proline rich 13	12	1.34	0.01892
IGFLR1	IGF like family receptor 1	19	1.82	0.01892
ECM2	extracellular matrix protein 2	9	0.42	0.01892
SCTR	secretin receptor	2	2.51	0.01892
STAC	SH3 and cysteine rich domain	3	2.10	0.01900
NPL	N-acetylneuraminate pyruvate lyase	1	1.86	0.01903
CHDH	choline dehydrogenase	3	1.79	0.01904
TMEM67	transmembrane protein 67	8	0.59	0.01904
QPRT	quinolinate phosphoribosyltransferase	16	1.62	0.01904
PTAFR	platelet activating factor receptor	1	1.71	0.01904
DHX32	DEAH-box helicase 32 (putative)	10	1.60	0.01918
PLBD1	phospholipase B domain containing 1	12	2.20	0.01918
SNAPC2	small nuclear RNA activating complex polypeptide 2	19	1.48	0.01918
CD68	CD68 molecule	17	1.88	0.01960
RGS12	regulator of G protein signaling 12	4	1.93	0.01992
BHLHE41	basic helix-loop-helix family member e41	12	2.06	0.02039
BCKDK	branched chain ketoacid dehydrogenase kinase	16	1.53	0.02039
MAOB	monoamine oxidase B	X	0.45	0.02072
CXCL16	C-X-C motif chemokine ligand 16	17	1.82	0.02072
HSD3B7	hydroxy-delta-5-steroid dehydrogenase, 3 beta- and steroid delta-isomerase 7	16	1.75	0.02084
CEP126	centrosomal protein 126	11	0.71	0.02086

DNASE2B	deoxyribonuclease 2 beta	1	2.55	0.02095
RAB38	RAB38, member RAS oncogene family	11	1.56	0.02108
TBC1D7	TBC1 domain family member 7	6	1.52	0.02108
CNN3	calponin 3	1	0.69	0.02108
ATG16L2	autophagy related 16 like 2	11	1.65	0.02121
TBXAS1	thromboxane A synthase 1	7	1.71	0.02121
TANGO2	transport and golgi organization 2 homolog	22	1.60	0.02171
SCNN1G	sodium channel epithelial 1 gamma subunit	16	0.49	0.02194
MSRA	methionine sulfoxide reductase A	8	1.51	0.02225
KCTD3	potassium channel tetramerization domain containing 3	1	0.68	0.02232
UPP1	uridine phosphorylase 1	7	2.05	0.02286
RNF175	ring finger protein 175	4	1.16	0.02286
TNFAIP2	TNF alpha induced protein 2	14	1.81	0.02300
TMEM52B	transmembrane protein 52B	12	1.28	0.02300
ADPGK	ADP dependent glucokinase	15	1.47	0.02300
PFKFB4	6-phosphofructo-2-kinase/fructose-2,6-biphosphatase 4	3	1.63	0.02312
NADSYN1	NAD synthetase 1	11	1.47	0.02364
CD52	CD52 molecule	1	2.44	0.02388
TRPV2	transient receptor potential cation channel subfamily V member 2	17	1.98	0.02388
PRAM1	PML-RARA regulated adaptor molecule 1	19	1.90	0.02388
PPARG	peroxisome proliferator activated receptor gamma	3	2.14	0.02406
SLC25A6	solute carrier family 25 member 6	X;Y	1.48	0.02406
WFDC21P	WAP four-disulfide core domain 21, pseudogene	17	2.24	0.02422
GPRIN3	GPRIN family member 3	4	1.60	0.02476
PFN2	profilin 2	3	0.55	0.02488
ST6GALNAC5	ST6 N-acetylgalactosaminide alpha-2,6-sialyltransferase 5	1	0.50	0.02488
SLITRK2	SLIT and NTRK like family member 2	X	0.78	0.02488
GBA	glucosylceramidase beta	1	1.66	0.02488
CLEC5A	C-type lectin domain containing 5A	7	1.90	0.02504
PIH1D2	PIH1 domain containing 2	11	0.75	0.02504
TPP1	tripeptidyl peptidase 1	11	1.76	0.02518
SHISAL2A	shisa like 2A	1	1.36	0.02518
PNPLA6	patatin like phospholipase domain containing 6	19	1.86	0.02518
NQO1	NAD(P)H quinone dehydrogenase 1	16	1.66	0.02518
SLC46A3	solute carrier family 46 member 3	13	1.62	0.02518
FCRLB	Fc receptor like B	1	1.79	0.02518
MYL9	myosin light chain 9	20	0.53	0.02518
SOX2	SRY-box 2	3	0.44	0.02518
USP30	ubiquitin specific peptidase 30	12	1.28	0.02522

ADAMTSL4	ADAMTS like 4	1	1.72	0.02522
MYBPHL	myosin binding protein H like	1	1.58	0.02522
MCF2L2	MCF.2 cell line derived transforming sequence-like 2	3	1.20	0.02522
NAPRT	nicotinate phosphoribosyltransferase	8	1.67	0.02566
GEMIN4	gem nuclear organelle associated protein 4	17	1.54	0.02629
SPARC	secreted protein acidic and cysteine rich	5	0.44	0.02630
LILRA6	leukocyte immunoglobulin like receptor A6	19	1.90	0.02661
ANTXR1	anthrax toxin receptor 1	2	0.50	0.02661
AP2S1	adaptor related protein complex 2 sigma 1 subunit	19	1.40	0.02673
TALDO1	transaldolase 1	11	1.52	0.02741
CYBB	cytochrome b-245 beta chain	X	2.26	0.02754
ZNF135	zinc finger protein 135	19	0.65	0.02754
GM2A	GM2 ganglioside activator	5	1.99	0.02761
ATG7	autophagy related 7	3	1.66	0.02786
KCTD5	potassium channel tetramerization domain containing 5	16	1.71	0.02801
MCEMP1	mast cell expressed membrane protein 1	19	2.46	0.02856
GPC3	glypican 3	X	0.37	0.02874
HK2	hexokinase 2	2	2.06	0.02874
CDCP1	CUB domain containing protein 1	3	1.76	0.02874
PTPMT1	protein tyrosine phosphatase, mitochondrial 1	11	1.60	0.02874
G6PD	glucose-6-phosphate dehydrogenase	X	1.60	0.02922
OAZ1	ornithine decarboxylase antizyme 1	19	1.36	0.02931
ADAP2	ArfGAP with dual PH domains 2	17	1.61	0.02951
TTLL11	tubulin tyrosine ligase like 11	9	1.17	0.02954
MPL	MPL proto-oncogene, thrombopoietin receptor	1	0.80	0.02958
PLA2G15	phospholipase A2 group XV	16	1.77	0.02959
SLC25A19	solute carrier family 25 member 19	17	1.67	0.02964
GPD1	glycerol-3-phosphate dehydrogenase 1	12	2.06	0.02964
IMPDH1	inosine monophosphate dehydrogenase 1	7	1.93	0.02964
CRIP2	cysteine rich protein 2	14	0.47	0.02964
ALKBH6	alkB homolog 6	19	1.30	0.02964
SDSL	serine dehydratase like	12	1.72	0.02975
CSTB	cystatin B	21	1.77	0.02980
LRRC34	leucine rich repeat containing 34	3	0.77	0.03009
GNAZ	G protein subunit alpha z	22	0.85	0.03009
GPX1	glutathione peroxidase 1	3	1.70	0.03009
GPN2	GPN-loop GTPase 2	1	1.42	0.03015
CATSPER1	cation channel sperm associated 1	11	1.61	0.03029
DHRS9	dehydrogenase/reductase 9	2	1.95	0.03132
ANGPTL5	angiopoietin like 5	11	0.69	0.03133

FCER1G	Fc fragment of IgE receptor Ig	1	2.14	0.03133
NPNT	nephronectin	4	0.64	0.03153
LINC01091	long intergenic non-protein coding RNA 1091	4	0.56	0.03153
ANGEL1	angel homolog 1	14	1.47	0.03207
TREM2	triggering receptor expressed on myeloid cells 2	6	2.17	0.03207
NCF4	neutrophil cytosolic factor 4	22	1.72	0.03238
SHKBP1	SH3KBP1 binding protein 1	19	1.41	0.03269
ATP6V0D1	ATPase H+ transporting V0 subunit d1	16	1.83	0.03271
C6orf118	chromosome 6 open reading frame 118	6	0.75	0.03271
SIGLEC7	sialic acid binding Ig like lectin 7	19	1.25	0.03271
ATP6V1G2	ATPase H+ transporting V1 subunit G2	6	0.78	0.03274
NCLN	nicalin	19	1.73	0.03302
C19orf54	chromosome 19 open reading frame 54	19	1.49	0.03302
LRRC8D	leucine rich repeat containing 8 VRAC subunit D	1	1.40	0.03302
LOC730101	uncharacterized LOC730101	6	0.78	0.03302
MAMDC2	MAM domain containing 2	9	0.42	0.03302
PGD	phosphogluconate dehydrogenase	1	1.92	0.03302
APMAP	adipocyte plasma membrane associated protein	20	1.47	0.03305
SPOCD1	SPOC domain containing 1	1	2.43	0.03305
CMTM7	CKLF like MARVEL transmembrane domain containing 7	3	1.57	0.03431
GCNT1	glucosaminyl (N-acetyl) transferase 1, core 2	9	1.62	0.03431
IRF8	interferon regulatory factor 8	16	1.80	0.03431
AGPS	alkylglycerone phosphate synthase	2	1.56	0.03431
HPS4	HPS4, biogenesis of lysosomal organelles complex 3 subunit 2	22	1.26	0.03450
TANC1	tetratricopeptide repeat, ankyrin repeat and coiled-coil containing 1	2	0.61	0.03471
CCDC36	coiled-coil domain containing 36	3	1.14	0.03489
MARCO	macrophage receptor with collagenous structure	2	2.88	0.03489
IFRD2	interferon related developmental regulator 2	3	1.45	0.03489
MBNL2	muscleblind like splicing regulator 2	13	0.62	0.03489
TYSND1	trypsin domain containing 1	10	1.63	0.03541
AXL	AXL receptor tyrosine kinase	19	1.68	0.03559
VPS33A	VPS33A, CORVET/HOPS core subunit	12	1.26	0.03622
TMEM97	transmembrane protein 97	17	1.74	0.03622
CTC1	CST telomere replication complex component 1	17	1.54	0.03622
KCNQ1	potassium voltage-gated channel subfamily Q member 1	11	1.26	0.03628
TYROBP	TYRO protein tyrosine kinase binding protein	19	1.88	0.03634

GCDH	glutaryl-CoA dehydrogenase	19	1.34	0.03663
AGRP	agouti related neuropeptide	16	3.01	0.03663
TTC39B	tetratricopeptide repeat domain 39B	9	1.53	0.03663
UBR3	ubiquitin protein ligase E3 component n-recognin 3 (putative)	2	1.28	0.03663
SGF29	SAGA complex associated factor 29	16	1.46	0.03663
ZMYND15	zinc finger MYND-type containing 15	17	1.78	0.03663
TMEM47	transmembrane protein 47	X	0.49	0.03663
C8orf48	chromosome 8 open reading frame 48	8	0.80	0.03663
CENPV	centromere protein V	17	1.51	0.03663
MAN2B1	mannosidase alpha class 2B member 1	19	1.72	0.03663
TMEM53	transmembrane protein 53	1	1.41	0.03663
ZDHHC23	zinc finger DHHC-type containing 23	3	0.71	0.03667
AFDN	afadin, adherens junction formation factor	6	0.45	0.03675
LRRC28	leucine rich repeat containing 28	15	1.51	0.03675
CCDC88B	coiled-coil domain containing 88B	11	1.33	0.03716
TSPAN32	tetraspanin 32	11	1.81	0.03777
SHC4	SHC adaptor protein 4	15	0.86	0.03779
RIN3	Ras and Rab interactor 3	14	1.34	0.03790
LAMP1	lysosomal associated membrane protein 1	13	1.49	0.03790
CENPN	centromere protein N	16	1.48	0.03793
TSPO	translocator protein	22	1.54	0.03804
FAM118B	family with sequence similarity 118 member B	11	1.30	0.03820
KMO	kynurenine 3-monooxygenase	1	1.56	0.03820
ANO1	anoctamin 1	11	0.38	0.03820
MANBA	mannosidase beta	4	1.55	0.03835
SLC6A12	solute carrier family 6 member 12	12	1.64	0.03835
PKD2L1	polycystin 2 like 1, transient receptor potential cation channel	10	1.72	0.03915
DCXR	dicarbonyl and L-xylulose reductase	17	1.63	0.03925
ZNF438	zinc finger protein 438	10	1.35	0.03925
SEMA3B	semaphorin 3B	3	0.57	0.03925
LDHD	lactate dehydrogenase D	16	1.49	0.03925
TTLL4	tubulin tyrosine ligase like 4	2	1.29	0.03925
SMARCA1	SWI/SNF related, matrix associated, actin dependent regulator of chromatin, subfamily a, member 1	X	0.67	0.03927
NADK	NAD kinase	1	1.35	0.03927
GMIP	GEM interacting protein	19	1.55	0.03942
CAMSAP1	calmodulin regulated spectrin associated protein 1	9	1.34	0.03973
LILRA2	leukocyte immunoglobulin like receptor A2	19	2.01	0.03986
DPP7	dipeptidyl peptidase 7	9	1.91	0.04012
OPTN	optineurin	10	0.69	0.04036
ACAT2	acetyl-CoA acetyltransferase 2	6	1.64	0.04036

HDDC2	HD domain containing 2	6	1.42	0.04044
TWF2	twinfilin actin binding protein 2	3	1.58	0.04189
AP1S3	adaptor related protein complex 1 sigma 3 subunit	2	0.78	0.04190
PSD4	pleckstrin and Sec7 domain containing 4	2	1.39	0.04295
PLA2G4E	phospholipase A2 group IVE	15	1.35	0.04295
UBE2I	ubiquitin conjugating enzyme E2 I	16	1.42	0.04307
HP	haptoglobin	16	4.49	0.04318
NCEH1	neutral cholesterol ester hydrolase 1	3	1.99	0.04318
KAT8	lysine acetyltransferase 8	16	1.61	0.04318
BBOF1	basal body orientation factor 1	14	0.52	0.04321
CDO1	cysteine dioxygenase type 1	5	0.44	0.04321
SYNGR2	synaptogyrin 2	17	1.51	0.04321
SYK	spleen associated tyrosine kinase	9	1.56	0.04321
SLC11A1	solute carrier family 11 member 1	2	2.56	0.04321
HDGFL3	HDGF like 3	15	0.60	0.04328
SLC25A40	solute carrier family 25 member 40	7	1.34	0.04328
PMEPA1	prostate transmembrane protein, androgen induced 1	20	0.61	0.04366
ACOT4	acyl-CoA thioesterase 4	14	1.70	0.04440
WDFY4	WDFY family member 4	10	1.33	0.04440
RUNX1	runt related transcription factor 1	21	1.55	0.04440
RPS2P32	ribosomal protein S2 pseudogene 32	7	1.64	0.04440
HMGCR	3-hydroxy-3-methylglutaryl-CoA reductase	5	1.52	0.04440
MGAT4A	mannosyl (alpha-1,3-)-glycoprotein beta-1,4-N-acetylglucosaminyltransferase, isozyme A	2	1.50	0.04440
IKBKE	inhibitor of nuclear factor kappa B kinase subunit epsilon	1	1.42	0.04440
MON1B	MON1 homolog B, secretory trafficking associated	16	1.26	0.04468
KIF21A	kinesin family member 21A	12	0.59	0.04468
STX2	syntaxin 2	12	0.66	0.04473
ERO1A	endoplasmic reticulum oxidoreductase 1 alpha	14	1.41	0.04516
NLRC4	NLR family CARD domain containing 4	2	1.64	0.04562
MYO9B	myosin IXB	19	1.60	0.04562
MAPKAPK3	mitogen-activated protein kinase-activated protein kinase 3	3	1.63	0.04562
FAM161A	family with sequence similarity 161 member A	2	0.81	0.04562
OGFRL1	opioid growth factor receptor like 1	6	1.40	0.04662
PCNX1	pecanex homolog 1	14	1.53	0.04673
SLC29A3	solute carrier family 29 member 3	10	1.60	0.04678
KLHL8	kelch like family member 8	4	1.51	0.04685
ADPRM	ADP-ribose/CDP-alcohol diphosphatase, manganese dependent	17	1.30	0.04722

MGAT1	mannosyl (alpha-1,3-)-glycoprotein beta-1,2-N-acetylglucosaminyltransferase	5	1.62	0.04768
EMILIN2	elastin microfibril interfacer 2	18	1.84	0.04773
ITGAL	integrin subunit alpha L	16	1.64	0.04787
IMP4	IMP4, U3 small nucleolar ribonucleoprotein	2	1.51	0.04842
SKA2	spindle and kinetochore associated complex subunit 2	17	0.67	0.04842
KCNAB1	potassium voltage-gated channel subfamily A member regulatory beta subunit 1	3	2.12	0.04914
CDHR3	cadherin related family member 3	7	0.26	0.04923
SCAMP2	secretory carrier membrane protein 2	15	1.44	0.04923
MSR1	macrophage scavenger receptor 1	8	2.24	0.04944
PI4K2A	phosphatidylinositol 4-kinase type 2 alpha	10	1.31	0.04966
DEF6	DEF6, guanine nucleotide exchange factor	6	1.50	0.04966
GRB2	growth factor receptor bound protein 2	17	1.44	0.04966
LPCAT1	lysophosphatidylcholine acyltransferase 1	5	1.45	0.04966
ROBO2	roundabout guidance receptor 2	3	0.63	0.04975
BCHE	butyrylcholinesterase	3	0.42	0.04975
STOX1	storkhead box 1	10	0.68	0.04975
TREM1	triggering receptor expressed on myeloid cells 1	6	2.30	0.04975
AKR1A1	aldo-keto reductase family 1 member A1	1	1.43	0.04975
GALNT12	polypeptide N-acetylgalactosaminyltransferase 12	9	1.66	0.04975
CARD9	caspase recruitment domain family member 9	9	1.60	0.04983

[a] Up-regulated genes have fold change >1, and down-regulated genes have fold change <1.

[b] *P*-values corrected for multiple testing using the Benjamini-Hochberg method to obtain the false discovery rate (FDR); the threshold for statistical significance was set at FDR <0.05.
