# Supplementary material for: Cigarette smoke alters the transcriptome of non-involved lung tissue in lung adenocarcinoma patients
Source: Sci Rep. 2019 Sep 10;9:13039. doi: 10.1038/s41598-019-49648-2 (PMC6736939; doi:10.1038/s41598-019-49648-2)
Supplement: Supplementary file 2 — Supplementary Table 2 [file 41598_2019_49648_MOESM2_ESM.pdf]

Cigarette smoke alters the transcriptome of non-involved lung tissue in lung adenocarcinoma patients

Giulia Pintarelli, Sara Noci, Davide Maspero, Angela Pettinicchio, Matteo Dugo, Loris De Cecco, Matteo Incarbone, Davide Tosi, Luigi Santambrogio, Tommaso A. Dragani, Francesca Colombo

Supplementary Table 2. Networks identified by gene interaction analysis performed with Ingenuity Pathway Analysis software

Molecules in Network	Score	Molecules	Top Diseases and Functions
AFDN, BCKDK, BCR (complex), BHLHE41, c-Src, CD3, CDCP1, CLEC5A, DOCK2, EGLN, EIF4EBP1, ERK1/2, FCER1G, GPNMB, GRB2, HK2, IGF2R, Igm, KCTD3, LAMP1, LAT2, p85 (pik3r), PFKFB4, PTPN6, RBP4, RPS2, RRAGD, SEMA3B, SHC4, SIGLEC7, SYK, SYNE2, TALDO1, TCR, TYROBP	40	27	Cell Death and Survival, Cellular Compromise, Cell-To-Cell Signaling and Interaction
Akt, ALOX5, ALOX5AP, Ap1, ATP6V0D1, AXL, CD68, CD1A, CDH15, CYP27A1, ELF5, ERK, estrogen receptor, G6PD, GBA, GPX1, Histone h4, Hsp27, Hsp90, IgG, IL1A, KAT8, KLK7, LDL, Mek, MYL9, NCF4, OSCAR, P38 MAPK, PCDH20, PPARG, PPARGC1A, RUNX1, SPARC, UBE2I	33	24	Immunological Disease, Dermatological Diseases and Conditions, Inflammatory Disease
BTK, CARD9, CD300LF, CFTR, CXCL16, CYBB, FABP5, Fcgr2, Fcgr3, FGR, Fibrinogen, Focal adhesion kinase, FUCA1, HMGCR, IFN Beta, IKBKE, IKK (complex), IL12 (complex), Interferon alpha, IRF8, ITGAL, ITGAX, ITGB2, MSR1, NFkB (complex), NLRC4, OLR1, OPTN, PARVG, PGD, PI3K (complex), Pkc(s), PTAFR, SLC9A3R1, Vegf	31	23	Infectious Diseases, Cellular Movement, Immune Cell Trafficking
ACTB, ANO1, ANTXR1, BIRC7, CD52, COL18A1, DEF6, DHRS9, F3, FBP1, FGFR1, FN1, GCHFR, GCNT1, IGFBP5, ITGA4, ITGB2, KDR, LGALS1, LGALS3, LRRC34, MAOB, MMP7, NRP1, PLPP3, POU2F2, PTPRC, S100A6, SLC11A1, SMARCA4, SPOCD1, ST6GALNAC5, TREM2, VCL, ZDHHC23	20	17	Cardiovascular System Development and Function, Organismal Development, Visual System Development and Function
ADORA2B, ASAH1, CCL17, CFB, DCSTAMP, DCXR, DPP7, ELOVL7, F2RL1, GCLM, GEMIN4, GRB2, HEXA, HEXB, HSD3B7, HTRA4, IL15RA, IL36RN, KANK1, KCNAB1, KIF21A, LAMP3, MAFF, MCF2L2, MOAP1, PIN1, PRSS21, SLAMF7, SPHK1, STAC, TBC1D7, TNF, TNIP3, TREM1, TSPO	20	17	Lipid Metabolism, Molecular Transport, Small Molecule Biochemistry
ABCG2, ABL1, ABLIM1, ACSL3, ATP6AP1, CAV2, CYP1B1, ELF5, ESR1, FDFT1, GGA3, GPS2, HDGFL3, HISTONE, HMGCS1, HVCN1, ITCH, LAPTM5, MAP4K1, MARCO, MBNL2, NPPB, NRIP1, OLR1, P2RX4, PFN2, PGR, PSMD4, QPRT, SLC2A3, STAT5A, SYVN1, TMEM97, TP53, TTLL11	18	16	Organ Development, Reproductive System Development and Function, Tissue Development
ALPK1, ANGEL1, AR, BRD2, CHD4, DEFB4A/DEFB4B, EMILIN2, ERO1A, ETS1, FLI1, HPS1, HPS4, IFIT2, IGF2BP3, IRF8, ITGB2, JAK2, LMNB1, MMD, MPL, NCEH1, NPC1, NQO1, NUPR1, PARP1, RAB32, RAB38, SFTPA1, SH2B3, SIRPA,	18	16	Cellular Movement, Hematological System Development and Function, Immune Cell Trafficking

SKA2, SRC (family), STAT1, TBXAS1, UBIAD1			
ADGRF1, CD163, COL1A1, COLGALT1, CXCL8, elastase, FABP5, FGB, FGG, GMIP, GUSB, HAMP, HP, HSPB2, HSPB3, IL6, IL1B, IL1R2, ITGAX, ITPA, KCNQ1, LILRA2, Lymphotoxin, MAML1, MAP3K7, NLRP12, Nr1h, SCTR, SGF29, SLC6A12, SYTL1, TBL1XR1, TEX14, THRA, TRAF6	18	16	Cell-To-Cell Signaling and Interaction, Cellular Growth and Proliferation, Skeletal and Muscular System Development and Function
BBOF1, BCAP31, CAMSAP1, CASP8, CBFA2T3, CCND1, CDO1, CENPN, CK1, CYCS, ECM2, FIG4, GATA4, HIC1, LIF, MGAT4A, MYO9B, PIKFYVE, PLAC1, POU2F1, PRKCD, PTCRA, RN7SK, SLC25A6, SNAPC1, SNAPC2, SNAPC3, SNAPC4, SOX2, SOX17, TCF4, TMEM47, TP53INP1, TRPV2, VAC14	15	14	Gene Expression, Carbohydrate Metabolism, Lipid Metabolism
ACAT2, BMI1, Cbp/p300, CBX4, CD44, CST5, EPCAM, EYA4, GADD45B, HK2, HNRNPA2B1, HSD17B14, KAT5, LINC01091, MAPKAPK3, MYC, MYO1A, PCSK9, PHC1, PIAS2, PSEN1, RING1, S1PR3, SCAMP2, SCNN1A, SCNN1B, SCNN1G, SLC25A19, SPINK5, STOX1, T, TAL1, TIAM1, TSPAN8, TWIST2	15	14	Cardiovascular Disease, Hereditary Disorder, Metabolic Disease
26s Proteasome, ACP5, ATG7, CAP2, CAPZA1, CASP4, CRIP2, CSTB, CYP1B1, DUSP14, FGA, FGB, FGG, FKBP15, FSH, GEM, GK, GNRHR, Histone h3, HSD11B1, HSD3B1, Lh, MAP4K4, MCOLN1, PHKA2, PI4K2A, PTPRE, RAB1A, RAPGEF3, RGS12, RGS16, RNA polymerase II, TLN1, TYRO3, UPP1	13	13	Developmental Disorder, Hematological Disease, Hereditary Disorder
ACSS2, C3, CCL3, CCL22, CD36, CD40, CD86, CMTM7, COL18A1, CXCL1, E2F3, EIF3E, EPAS1, FCER1G, GM2A, HGF, HK3, IL13, IL1B, ITGA6, JAK2, JAML, LILRB2, LTA4H, NCLN, PHLDA1, PLA2R1, PRR13, RIN2, SYNGR2, TGM2, THBS1, TMC6, TNFAIP2, TPP1	13	13	Cardiovascular System Development and Function, Cell-To-Cell Signaling and Interaction, Inflammatory Response
ADCY, AGRP, CDC25A, CDKN1A, CHDH, CHEK1, CSF1R, DCN, ELOA, EPB41L1, ERBB2, FANCE, FGFR3, FRMD3, HEY1, JAG1, KDM5B, LEP, let-7, MC3R, MCM4, MED1, MGAT1, miR-16-5p (and other miRNAs w/seed AGCAGCA), NRAS, NTRK1, PMEPA1, PURA, RAB31, RIN3, RNA polymerase II, SMARCA1, TOP2A, TOP3A, ZNF589	11	11	Cell Cycle, Cellular Growth and Proliferation, DNA Replication, Recombination, and Repair
ADIPOQ, AGPS, CCL3, CCL4, CCL20, CD86, CD163, CIITA, CXCL9, DDIT3, EBI3, EIF2AK3, GCDH, Hdac, HLA-DMA, IFNG, IL23, IL27, IL12B, IL17A, IL1A, IL1RN, IRF4, LPCAT1, MRC1, PDCD1, PTPMT1, RORC, SLC15A3, SOD2, SPP1, TAP1, TBX21, TNFRSF10B, USF1	5	7	Immunological Disease, Inflammatory Disease, Inflammatory Response
