# Supplementary material for: Cigarette smoke alters the transcriptome of non-involved lung tissue in lung adenocarcinoma patients
Source: Sci Rep. 2019 Sep 10;9:13039. doi: 10.1038/s41598-019-49648-2 (PMC6736939; doi:10.1038/s41598-019-49648-2)
Supplement: Supplementary file 3 — Supplementary Table 3 [file 41598_2019_49648_MOESM3_ESM.pdf]

Cigarette smoke alters the transcriptome of non-involved lung tissue in lung adenocarcinoma patients

Giulia Pintarelli, Sara Noci, Davide Maspero, Angela Pettinicchio, Matteo Dugo, Loris De Cecco, Matteo Incarbone, Davide Tosi, Luigi Santambrogio, Tommaso A. Dragani, Francesca Colombo

Supplementary Table 3. Genes differentially expressed between ever and never smokers in the present study (IT) and in the studies of Bosse et al. 2012 (Laval, GRN and UBC datasets) and Landi et al. 2008 (Landi). Data are sorted by log_2FC for IT.

Gene symbol	Gene name	Chr.	Log_2FC				
			IT	Laval	GRN	UBC	Landi
KMO	kynurenine 3-monooxygenase	1	0.65	0.91	0.77	0.64	0.76
CD1A	CD1a molecule	1	0.95	2.48	1.09	1.61	0.65
SPINK5	serine peptidase inhibitor, Kazal type 5	5	1.08	1.44	0.90	0.76	0.63
TREM2	triggering receptor expressed on myeloid cells 2	6	1.11	1.74	1.11	0.80	0.70
CYBB	cytochrome b-245 beta chain	X	1.17	1.09	0.87	0.61	0.88
DNASE2B	deoxyribonuclease 2 beta	1	1.35	2.11	1.64	1.32	0.76
FGG	fibrinogen gamma chain	4	4.99	3.25	1.71	1.38	2.44

FC, fold change (ratio of expression level in ever to never smokers)
