# Supplementary material for: Cigarette smoke alters the transcriptome of non-involved lung tissue in lung adenocarcinoma patients
Source: Sci Rep. 2019 Sep 10;9:13039. doi: 10.1038/s41598-019-49648-2 (PMC6736939; doi:10.1038/s41598-019-49648-2)
Supplement: Supplementary file 4 — Supplementary Table 4 [file 41598_2019_49648_MOESM4_ESM.pdf]

Cigarette smoke alters the transcriptome of non-involved lung tissue in lung adenocarcinoma patients

Giulia Pintarelli, Sara Noci, Davide Maspero, Angela Pettinicchio, Matteo Dugo, Loris De Cecco, Matteo Incarbone, Davide Tosi, Luigi Santambrogio, Tommaso A. Dragani, Francesca Colombo

Supplementary Table 4. Validation of the seven-gene signature of smoking in lung parenchyma from 582 non-cancer patients (GSE47460 dataset [1])

	Ever (n = 423) vs. never (n = 136) smokers [2]		Current (n = 23) vs. never (n = 136) smokers [3]	
**Gene symbol**	**Log_2 fold change**	**FDR [4]**	**Log_2 fold change**	**FDR**
CD1A	0.35	6.48E-01	1.41	1.02E-04
CYBB	0.05	1.00E+00	0.64	1.39E-04
DNASE2B	0.35	7.87E-01	1.84	3.35E-06
FGG	1.26	9.39E-03	2.19	6.91E-03
KMO	0.13	1.00E+00	0.67	4.87E-03
SPINK5	0.25	7.68E-01	0.37	3.45E-01
TREM2	0.22	8.27E-01	1.06	7.88E-05

[1] 254 patients with interstitial lung disease, 220 patients with chronic obstructive lung disease, and 108 persons who had surgery for a lung nodule but were found to be free of lung disease
[2] A total of nine differentially expressed genes (FDR < 0.05) was detected.
[3] A total of 1160 differentially expressed genes (FDR < 0.05) was detected.
[4] FDR, false discovery rate.
