# Supplementary material for: Cigarette smoke alters the transcriptome of non-involved lung tissue in lung adenocarcinoma patients
Source: Sci Rep. 2019 Sep 10;9:13039. doi: 10.1038/s41598-019-49648-2 (PMC6736939; doi:10.1038/s41598-019-49648-2)
Supplement: Supplementary file 5 — Supplementary Figure 1 [file 41598_2019_49648_MOESM5_ESM.pdf]

Cigarette smoke alters the transcriptome of non-involved lung tissue in lung adenocarcinoma patients

Giulia Pintarelli, Sara Noci, Davide Maspero, Angela Pettinicchio, Matteo Dugo, Loris De Cecco, Matteo Incarbone, Davide Tosi, Luigi Santambrogio, Tommaso A. Dragani, Francesca Colombo

Supplementary Figure 1

Supplementary Figure 1. Cellular composition of lung tissue samples estimated from gene expression data using the online tool xCell. The heatmap reports the normalized enrichment scores for 64 immune and stromal cell types (displayed on the right) for each patient (from purple, the lowest, to yellow, the highest enrichment scores; white color indicated no statistically significant enrichment, i.e. $P > 0.05$). Patients are sorted into ever and never smoker groups.

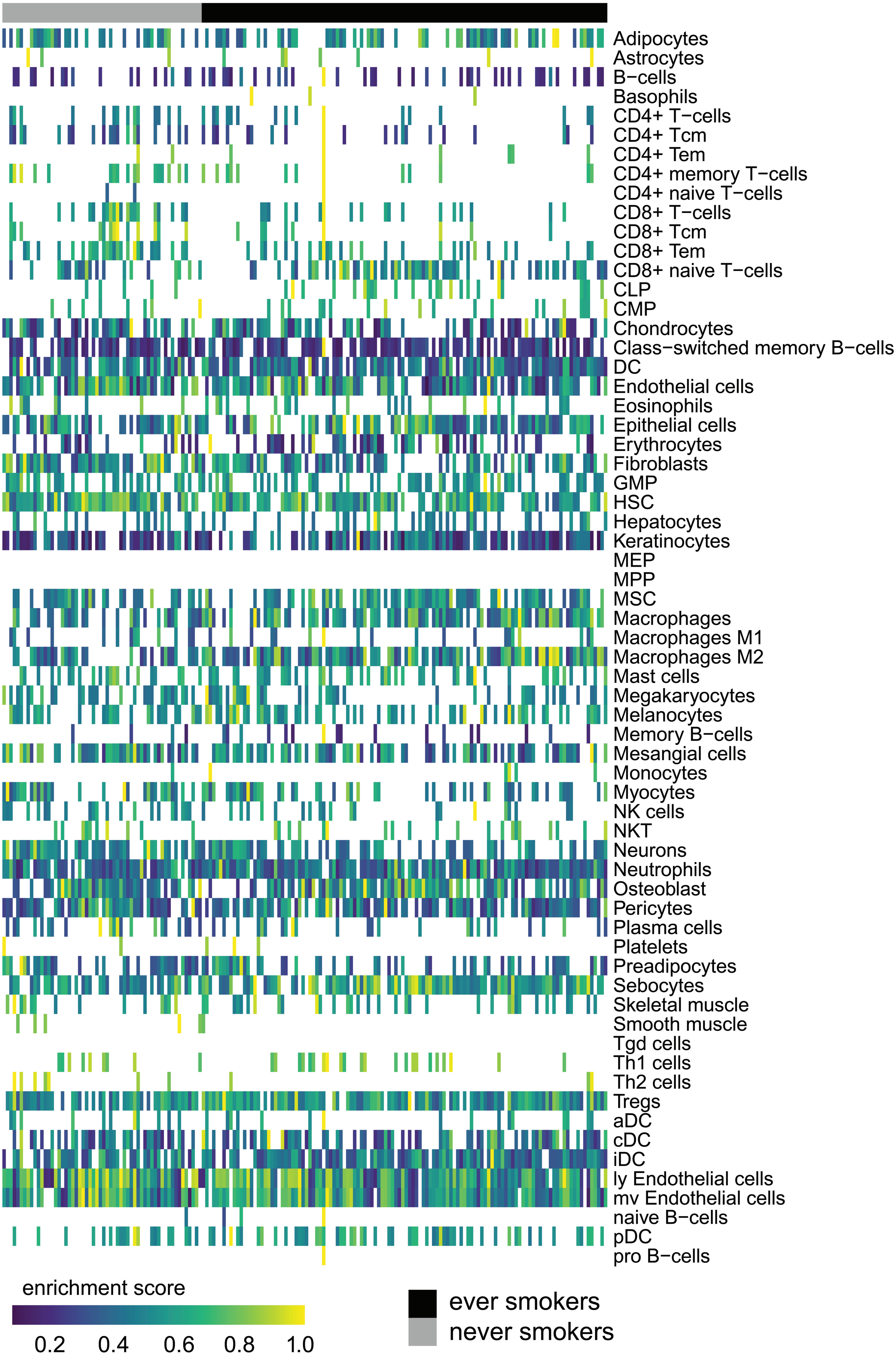
Adipocytes
Astrocytes
B−cells
Basophils
CD4+ T−cells
CD4+ Tcm
CD4+ Tem
CD4+ memory T−cells
CD4+ naive T−cells
CD8+ T−cells
CD8+ Tcm
CD8+ Tem
CD8+ naive T−cells
CLP
CMP
Chondrocytes
Class−switched memory B−cells
DC
Endothelial cells
Eosinophils
Epithelial cells
Erythrocytes
Fibroblasts
GMP
HSC
Hepatocytes
Keratinocytes
MEP
MPP
MSC
Macrophages
Macrophages M1
Macrophages M2
Mast cells
Megakaryocytes
Melanocytes
Memory B−cells
Mesangial cells
Monocytes
Myocytes
NK cells
NKT
Neurons
Neutrophils
Osteoblast
Pericytes
Plasma cells
Platelets
Preadipocytes
Sebocytes
Skeletal muscle
Smooth muscle
Tgd cells
Th1 cells
Th2 cells
Tregs
aDC
cDC
iDC
ly Endothelial cells
mv Endothelial cells
naive B−cells
pDC
pro B−cells
enrichment score
0.2
0.4
0.6
0.8
1.0
ever smokers
never smokers
