# Supplementary material for: Cigarette smoke alters the transcriptome of non-involved lung tissue in lung adenocarcinoma patients
Source: Sci Rep. 2019 Sep 10;9:13039. doi: 10.1038/s41598-019-49648-2 (PMC6736939; doi:10.1038/s41598-019-49648-2)
Supplement: Supplementary file 6 — Supplementary Figure 2 [file 41598_2019_49648_MOESM6_ESM.pdf]

Cigarette smoke alters the transcriptome of non-involved lung tissue in lung adenocarcinoma patients

Giulia Pintarelli, Sara Noci, Davide Maspero, Angela Pettinicchio, Matteo Dugo, Loris De Cecco, Matteo Incarbone, Davide Tosi, Luigi Santambrogio, Tommaso A. Dragani, Francesca Colombo

Supplementary Figure 2

Supplementary Figure 2. xCell immune (A) and stromal (B) enrichment scores in ever and never smokers. The line within each box represents the median value; upper and lower edges of each box are 75th and 25th percentiles, respectively; top and bottom whiskers indicate the maximum and minimum values, respectively. Kruskal-Wallis non-parametric test.

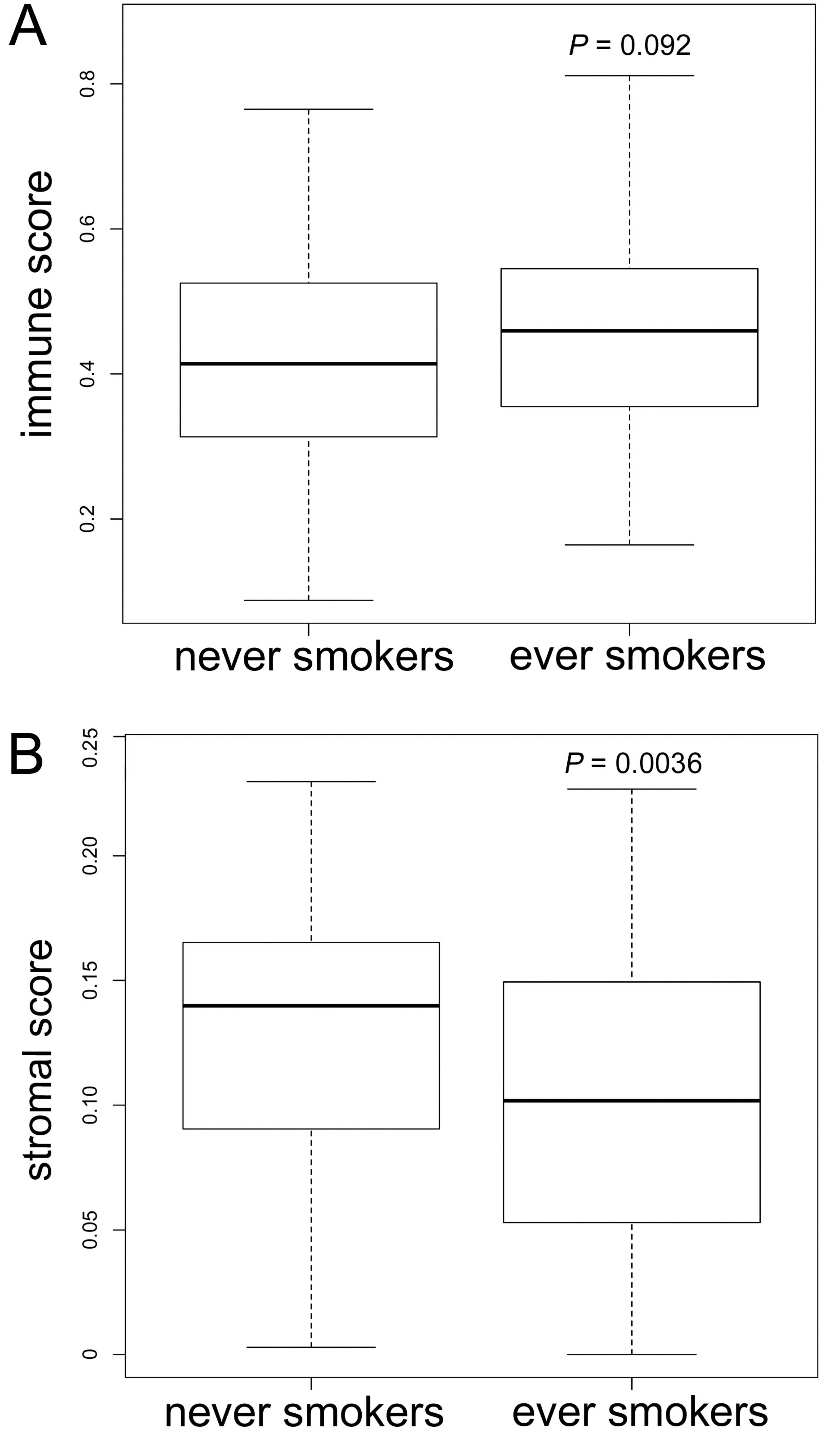

A
P = 0.092
0.8
0.6
0.4
0.2
immune score
never smokers
ever smokers
B
P = 0.0036
0.25
0.20
0.15
0.10
0.05
0
stromal score
never smokers
ever smokers
