# Supplementary material for: Cigarette smoke alters the transcriptome of non-involved lung tissue in lung adenocarcinoma patients
Source: Sci Rep. 2019 Sep 10;9:13039. doi: 10.1038/s41598-019-49648-2 (PMC6736939; doi:10.1038/s41598-019-49648-2)
Supplement: Supplementary file 7 — Supplementary Figure 3 [file 41598_2019_49648_MOESM7_ESM.pdf]

Cigarette smoke alters the transcriptome of non-involved lung tissue in lung adenocarcinoma patients

Giulia Pintarelli, Sara Noci, Davide Maspero, Angela Pettinicchio, Matteo Dugo, Loris De Cecco, Matteo Incarbone, Davide Tosi, Luigi Santambrogio, Tommaso A. Dragani, Francesca Colombo

Supplementary Figure 3

Supplementary Figure 3. Correlation between microarray data and quantitative PCR data from 27 ever smokers and 27 never smokers, regarding the seven genes differentially expressed between ever and never smokers in this study and in those of Bossé et al. and Landi et al. 4, 8. Regression lines are drawn in black, while the gray areas correspond to the 95% confidence interval. $P < 0.001$ for all correlations (Pearson's test).

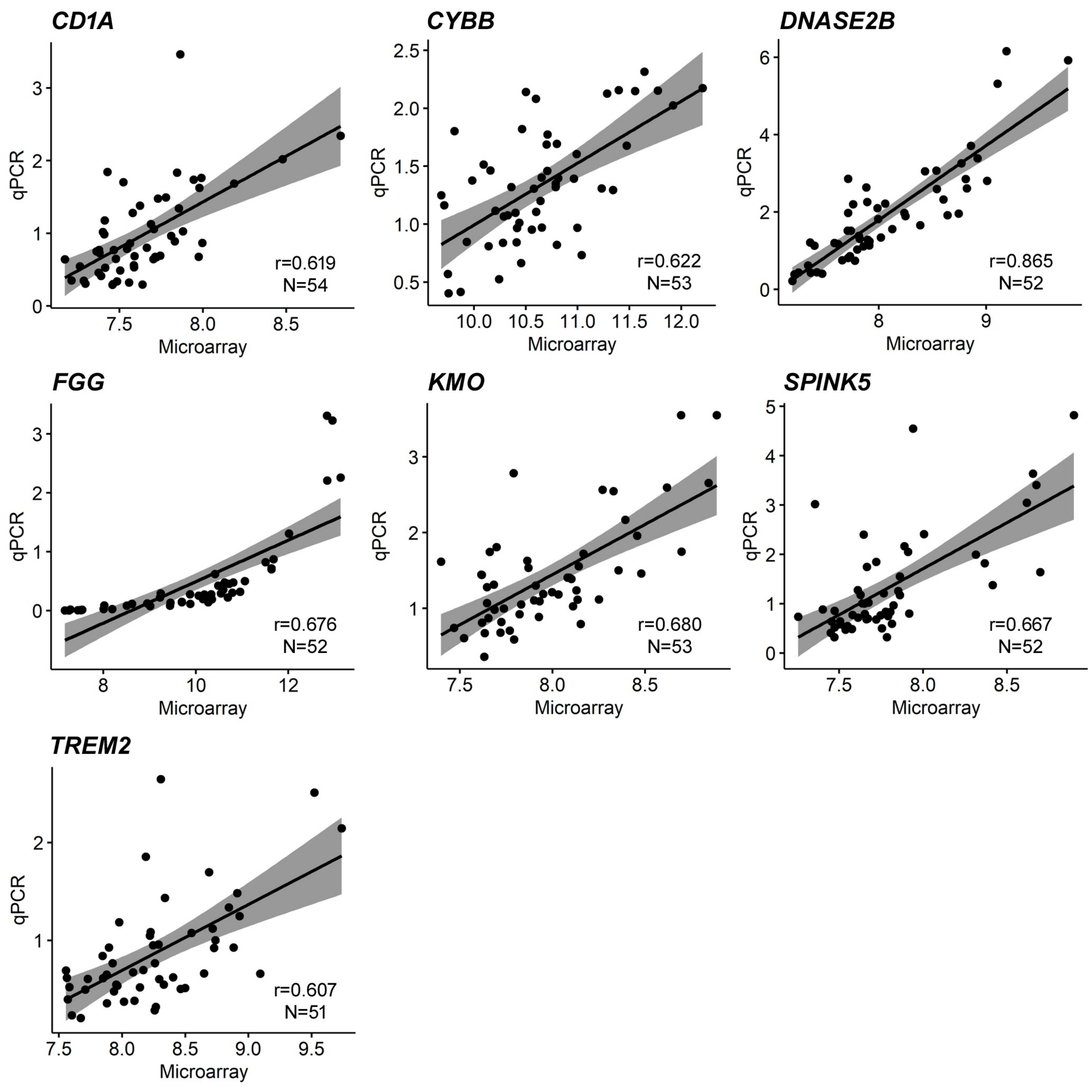

CD1A
r=0.619
N=54
CYBB
r=0.622
N=53
DNASE2B
r=0.865
N=52
FGG
r=0.676
N=52
KMO
r=0.680
N=53
SPINK5
r=0.667
N=52
TREM2
r=0.607
N=51
qPCR
Microarray
